# Gene transcription in bursting: a unified mode for realizing accuracy and stochasticity

**DOI:** 10.1111/brv.12452

**Published:** 2018-07-19

**Authors:** Yaolai Wang, Tengfei Ni, Wei Wang, Feng Liu

**Affiliations:** ^1^ National Laboratory of Solid State Microstructures, Department of Physics, and Collaborative Innovation Center of Advanced Microstructures Nanjing University Nanjing 210093 China; ^2^ School of Science Jiangnan University Wuxi 214122 China

**Keywords:** gene expression, MS2, PP7, ratchet model, multi‐scale model, continuum model, WLW model, temporal occupancy rate, frequency code, burst cluster

## Abstract

There is accumulating evidence that, from bacteria to mammalian cells, messenger RNAs (mRNAs) are produced in intermittent bursts – a much ‘noisier’ process than traditionally thought. Based on quantitative measurements at individual promoters, diverse phenomenological models have been proposed for transcriptional bursting. Nevertheless, the underlying molecular mechanisms and significance for cellular signalling remain elusive. Here, we review recent progress, address the above issues and illuminate our viewpoints with simulation results. Despite being widely used in modelling and in interpreting experimental data, the traditional two‐state model is far from adequate to describe or infer the molecular basis and stochastic principles of transcription. In bacteria, DNA supercoiling contributes to the bursting of those genes that express at high levels and are topologically constrained in short loops; moreover, low‐affinity *cis*‐regulatory elements and unstable protein complexes can play a key role in transcriptional regulation. Integrating data on the architecture, kinetics, and transcriptional input–output function is a promising approach to uncovering the underlying dynamic mechanism. For eukaryotes, distinct bursting features described by the multi‐scale and continuum models coincide with those predicted by four theoretically derived principles that govern how the transcription apparatus operates dynamically. This consistency suggests a unified framework for comprehending bursting dynamics at the level of the structural and kinetic basis of transcription. Moreover, the existing models can be unified by a generic model. Remarkably, transcriptional bursting enables regulatory information to be transmitted in a digital manner, with the burst frequency representing the strength of regulatory signals. Such a mode guarantees high fidelity for precise transcriptional regulation and also provides sufficient randomness for realizing cellular heterogeneity.

## I. INTRODUCTION

Like a coin with two opposite sides, gene transcription exhibits duality. On the one hand, it is an accurate process. When and at what rate to transcribe a gene is subject to precise regulation, and accuracy is essential for cell fitness and development (Brivanlou & Darnell, 2002; Blake *et al*., 2003; Gregor *et al*., 2007; Fuda, Ardehali, & Lis, 2009; Weake & Workman, 2010; Wang, Liu, & Wang, 2012; Senecal *et al*., 2014). On the other hand, it is a stochastic process. Randomness is an inherent nature of biomolecular interactions, and the resulting heterogeneity is indispensible for cell differentiation and survival (Kussell & Leibler, 2005; Eldar & Elowitz, 2010; Torres‐Padilla & Chambers, 2014; Buettner *et al*., 2015; Dueck, Eberwine, & Kim, 2016). It is challenging to comprehend such a contradiction, especially given that messenger RNAs (mRNAs) are widely found to be generated in discontinuous bursts (Chubb *et al*., 2006; Raj *et al*., 2006; Chubb & Liverpool, 2010; Suter *et al*., 2011; Sanchez & Golding, 2013; Chong *et al*., 2014).

Traditional biochemical technologies assayed gene transcription in millions of cells simultaneously, leaving an impression that mRNAs were produced at a continuous and smooth rate. Taking into account the stochasticity of molecular interactions, the transcription rate of a gene (defined as the number of transcripts produced per unit time) was believed to fluctuate slightly around an average (Raj & van oudenaarden, 2008; Lenstra *et al*., 2016). Thus, mRNAs were thought to be produced with a constant probability per unit time, namely *via* a Poisson process.

**Figure 1:**
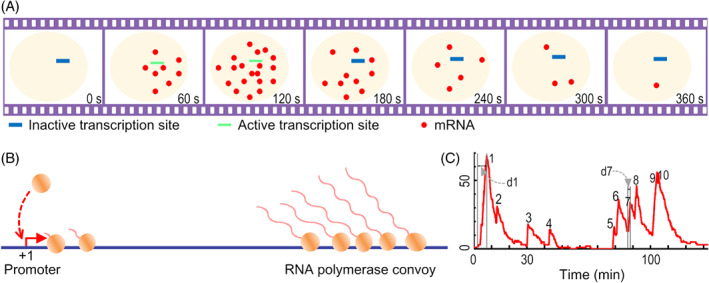
Schematic of transcriptional bursting. (A) A burst of messenger RNAs (mRNAs) is produced in a short time period with the gene active. (B) During each active period, RNA polymerases successively escape from the promoter and elongate as a convoy, leading to a burst. Two convoys are shown separately in generation and elongation. (C) A sample time series of the number of cellular mRNAs. Ten bursts are shown, with the duration of the first and seventh bursts denoted by d1 and d7.

Measurements in individual cells instead revealed that transcription occurs in intermittent bursts and that such bursting is ubiquitous from bacteria to mammalian cells (Golding *et al*., 2005; Chubb *et al*., 2006; Raj *et al*., 2006; Tripathi & Chowdhury, 2008; Suter *et al*., 2011; Sanchez & Golding, 2013; Chong *et al*., 2014; Corrigan *et al*., 2016; Fukaya, Lim, & Levine, 2016; Tantale *et al*., 2016). By labelling individual mRNAs using single‐molecule fluorescence *in situ* hybridization (smFISH), it was observed that a number of mRNAs from a gene emerge suddenly and then disappear gradually during a short time window (Raj *et al*., 2006; Battich, Stoeger, & Pelkmans, 2013; Larson *et al*., 2013; Buxbaum, Haimovich, & Singer, 2015; Lenstra *et al*., 2016) (Fig. 1A). To visualize transcription initiation directly, an array of a specific sequence is inserted after the promoter (Bertrand *et al*., 1998; Lim & Peabody, 2002; Chubb *et al*., 2006; Hocine *et al*., 2013). The transcribed sequence forms a stem loop, which binds the bacteriophage coat protein MS2 or PP7. The nascent mRNAs can thus be tracked in real time using time‐lapse fluorescence microscopy. It was found that many RNA polymerases successively escape from the promoter within several minutes, resulting in a convoy of closely spaced polymerases that elongates along the DNA and ultimately releases a burst of mRNAs (Tantale *et al*., 2016) (Fig. 1B). After the launch of this polymerase convoy, the promoter enters a refractory period without transcriptional activity.

In characterizing the time series of the number of cellular mRNAs, the duration of a burst is defined as its rising period that corresponds to the time window of synthesizing transcripts (Fig. 1C). Usually, the duration of a burst is no more than several minutes. The refractory period between two successive bursts ranges from seconds to tens of minutes or even longer. The magnitude of a burst can be up to dozens of mRNAs. The burst duration and magnitude are collectively termed burst size. Both the burst size and burst frequency (defined as the number of bursts per unit time) are potentially modulated by transcriptional activators.

Transcriptional bursting has been a hot topic of recent reviews (Boeger *et al*., 2015; Munsky & Neuert, 2015; Munsky, Fox, & Neuert, 2015; Reinius & Sandberg, 2015; Lenstra *et al*., 2016; Bressloff, 2017; Chubb, 2017; Hnisz *et al*., 2017; Nicolas, Phillips, & Naef, 2017; Yao, 2017). Here we focus on findings based on advanced quantitative investigations at individual alleles, aiming to connect the existing phenomenological models with the molecular mechanism of how transcription is dynamically orchestrated. We further elucidate that it is the frequency modulation that transmits information, which fulfills the accuracy and randomness requisites for transcriptional regulation.

## II. TWO‐STATE MODEL OF TRANSCRIPTIONAL BURSTING

**Figure 2:**
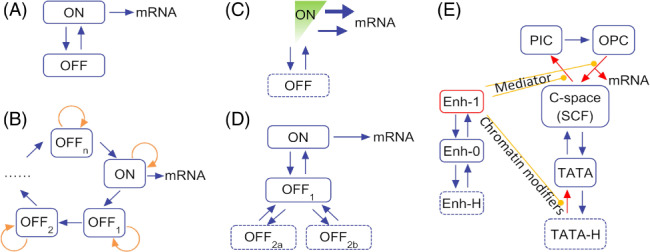
Models for transcriptional bursting. (A) Two‐state model. The gene has two alternative states, ON and OFF, whose lifetimes are usually assumed to obey two different exponential distributions. The mRNAs are produced *via* a Poisson process. (B) Ratchet model. A series of ordered sub‐OFF states exists. (C) Continuum model. Instead of a Poisson process, transcripts are generated at quasi‐continuous rates. (D) Multi‐scale model. Multiple layers of sub‐OFF states exist. (E) The Wang–Liu–Wang (WLW) model. The core promoter region is described as converting among five states, which are ‘being occupied by histones (TATA‐H)’, ‘naked (TATA)’, ‘being occupied by the C‐space (SCF), PIC or OPC’. The ‘C‐space’ refers to a clamp‐like space formed between the Mediator and the enhancer. The enhancer (Enh) has three states, i.e. being bound by histones (Enh‐H) or an activator (Enh‐1), or naked (Enh‐0). OPC, open complex; PIC, pre‐initiation complex; SCF, scaffold complex; TATA, TATA‐box.

Intuitively, transcriptional bursting can be characterized with a simple two‐state model (Fig. 2A), where the gene promoter switches between a transcriptionally active state (ON) and an inactive state (OFF) (Lionnet & Singer, 2012; Munsky, Neuert, & van Oudenaarden, 2012; Sanchez, Choubey, & Kondev, 2013; Albayrak *et al*., 2016; Xu *et al*., 2016). A burst of transcripts is generated in the ON state, whereas the OFF state is non‐permissive for transcription. The two‐state model is usually depicted by three parameters: activation rate *k*_on_ (reaction rate from the OFF to ON state), inactivation rate *k*_off_ (reaction rate from ON to OFF), and mRNA synthesis rate from the ON state *k*_m_. *k*_on_ is positively related to the concentration of transcriptional activators. Both *k*_off_ and *k*_m_ are constant, i.e. the duration of the ON state is exponentially distributed and mRNA synthesis is a Poisson process.

The original two‐state model was proposed to explain cell heterogeneity 25 years ago (Ko, 1992). It is widely used in the analysis of experimental data and theoretical simulation (see also the review by Sanchez *et al*., 2013). However, its application scope has not been well defined. Theoretically, it cannot be ruled out that the transcriptional dynamics described well by the two‐state model instead originate from other stochastic principles (Pedraza & Paulsson, 2008); indeed, advanced measurements have suggested much more complex models as discussed below. Results rooted in the two‐state model should therefore be interpreted with care.

## III. TRANSCRIPTIONAL BURSTING IN BACTERIA

In individual prokaryotic cells, the copy number of transcripts from a promoter is usually no more than 10 (Taniguchi *et al*., 2010), much lower than that in mammalian cells. Number fluctuation is thus conspicuous. Consequently, to uncover the principles that govern mRNA production necessitates collecting massive quantities of data *via* tracing the time series of transcription initiation events or changes in MS2/PP7‐labelled mRNAs. While this is realizable, it has not been done sufficiently. Nevertheless, it was confirmed that mRNA production is not a Poisson process but can be well fitted by the two‐state model (Golding *et al*., 2005; Taniguchi *et al*., 2010; So *et al*., 2011; Sanchez & Golding, 2013; Skinner *et al*., 2013; Chong *et al*., 2014).

The mechanism for transcriptional bursting in bacteria is largely unclear. However, for those genes that express at high levels and are topologically constrained in short loops (∼10000 base pairs on average), the bursting was revealed to be primarily caused by DNA supercoiling (Chong *et al.,* 2014; Levens & Larson, 2014; Ma & Wang, 2016). An elongating polymerase results in positive supercoiling ahead of it and negative supercoiling behind it. A long interval is required to relieve the positive supercoiling due to limited quantities of DNA gyrase; positive supercoiling thus gradually accumulates, slowing down the elongation of the polymerase. On the other hand, negative supercoiling can be rapidly relieved by topoisomerase 1A and possibly helps relieve the positive supercoiling caused by a posterior polymerase. Consequently, a convoy of polymerases stops elongating, and the transcription initiation is eventually suspended. Once the positive supercoiling is removed, the convoy proceeds rapidly. A newly launched polymerase that is far behind the convoy will lead another burst.

In‐depth exploration of the bursting mechanism requires determination of the pathways and kinetics of molecular interactions among the various regulatory proteins, σ factor, the polymerase, and *cis*‐regulatory elements (Pedraza & Paulsson, 2008; Friedman & Gelles, 2012; Gourse & Landick, 2012). This is challenging, especially when unstable protein complexes and unknown transient interactions are involved (Friedman & Gelles, 2012). A promising approach is to adopt a hybrid method that combines the data on molecular structure, molecular interaction kinetics, and transcriptional input–output function, as exemplified by a recent study which unraveled the transcriptional dynamics of the *glnAp2* promoter (Wang, Liu, & Wang, 2016).

**Figure 3:**
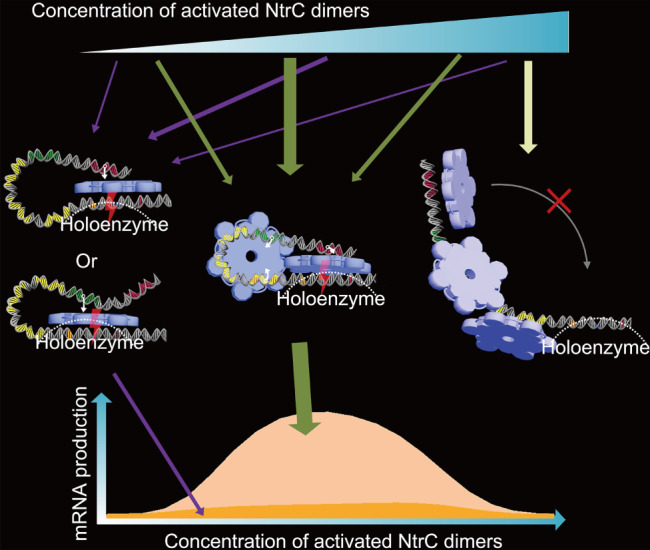
Molecular mechanisms for transcriptional regulation at the *glnAp2* promoter. A transcriptional activator nitrogen regulatory protein C (NtrC) hexamer (shown in blue) formed at the remote or proximal enhancer (separately coloured in red and green; the connection between an enhancer and an NtrC hexamer is denoted by white arrows) is able to catalyse the polymerase holoenzyme (outlined by a dashed line) at the core promoter (the −24, −12, and +1 sites are denoted in orange, blue, and red, respectively). For a wide range of NtrC concentrations, these two modes only contribute to a small proportion of messenger RNAs (mRNAs) produced, since it takes a long time for the hexamers to find the holoenzyme. In the third mode, there exists a DNA bridging—an NtrC hexamer connects the proximal enhancer to a low‐affinity site (the three low‐affinity sites are coloured in yellow). This bridging facilitates the hexamer at the remote enhancer to catalyse multiple rounds of transcription initiation. At very high concentrations of NtrC dimers, NtrC oligomers are formed at the low‐affinity sites, rendering the DNA rigid and turning the gene off.

Upstream of the *glnAp2* promoter, there are two enhancer sequences and three low‐affinity sequences for the binding of transcriptional activator nitrogen regulatory protein C (NtrC). In contrast with the traditional view that transcription initiation is stimulated by an NtrC hexamer simultaneously bound to the two enhancers, Wang *et al*. (2016) revealed that the initiation is stimulated by an NtrC hexamer at either enhancer (Fig. 3). Moreover, the main regulatory mode involves short‐lived DNA bridging, *via* which the proximal enhancer is connected to a low‐affinity site adjacent to the transcription start site (TTS). With the DNA bridging, the NtrC hexamer at the distal enhancer is in the vicinity of the TTS and thus immediately stimulates the polymerases one by one, leading to a burst. This work stresses the importance of low‐affinity sites, unstable protein complexes, and transient molecular interactions in orchestrating transcription.

## IV. TRANSCRIPTIONAL BURSTING IN EUKARYOTES

In higher eukaryotes, DNA is packaged by nucleosomes that consist of histones; both the DNA and histones are epigenetically modified (Catez, Ueda, & Bustin, 2006; Kouzarides, 2007; Coulon *et al*., 2013). Transcriptional progression begins with DNA demethylation, histone modification and nucleosome eviction. The transcription apparatus (TA), which is mainly composed of gene‐specific transcriptional activator(s), general transcription factors (TFs) including TFII‐B, ‐D, ‐E, ‐F, and ‐H, the Mediator complex, and RNA polymerase II (Pol II), assembles on the promoter to synthesise transcripts. The destruction of the TA may be followed by its reconstruction, or nucleosome recruitment and epigenetic reprogramming. Thus, the TA together with the chromatin determines transcriptional dynamics in eukaryotic cells.

For some genes, chromatin has a primary role in shaping transcriptional dynamics (Métivier *et al*., 2003; Métivier, Reid, & Gannon, 2006; Rybakova *et al*., 2015a). The promoter state, which is determined by how the promoter is chemically modified and how the associated proteins are structurally assembled and chemically modified, evolves sequentially due to successive occurrence of irreversible molecular reactions [e.g. the demethylation of DNA engages a series of reactions that orchestrate the oxidation of cytosine and base excision and repair (Nabel & Kohli, 2011)]. Moreover, the promoter state evolves cyclically, repeating the stages from histone eviction, then TA assembly and destruction, to chromatin recovery (Reid *et al*., 2003; Hager, McNally, & Misteli, 2009; Stavreva *et al*., 2009; Wang *et al*., 2014). Such irreversibility and periodicity were described by a ratchet model (Krasnov *et al*., 2016; Lemaire *et al*., 2006; Métivier *et al*., 2006; Wang *et al*., 2014) (Fig. 2B). Each cycle of the ratchet includes more than 100 molecular reactions, and most of the reactions involve chromatin modifications. Transcripts are produced during a short ‘ON’ period. Typical genes obeying such dynamics include the oestrogen receptor α‐regulated *pS2* gene in human cells and the copper receptor Ace1p‐regulated *CUP1* gene in yeast cells (Reid *et al*., 2003; Karpova *et al*., 2008; Wang *et al*., 2014). Once induced simultaneously by oestrogen or copper, a population of the promoters exhibits synchronized transcriptional activity for a relatively long time (Lemaire *et al*., 2006; Wang *et al*., 2014; Rybakova *et al*., 2015a).

For most genes, however, it seems that the promoter largely lacks histones or the chromatin can be easily opened. Extensive investigation revealed that the time series of transcriptional bursting is primarily shaped by the TA, whose gene specificity is defined by the *cis*‐regulatory elements and promoter sequence (Suter *et al*., 2011). Additionally, the duration of the ON state is exponentially distributed, while the OFF state cannot be described by one single step. Two novel models have been built to describe this type of promoter. One is the ‘continuum model’ based on a highly expressed housekeeping actin gene (Corrigan *et al*., 2016). The other is the ‘multi‐scale model’ based on the fully activated HIV‐1 gene (Tantale *et al*., 2016). High expression means that the promoter is largely evicted from histones, and thus the models accurately reflect the dynamics of the TA.

The continuum model has an OFF state and a special ON state (Corrigan *et al*., 2016) (Fig. 2C). During the ON period, transcripts are not generated *via* a Poisson process as assumed by the two‐state model. The time intervals between two successive initiation events range from several seconds to more than ten seconds. These intervals do not obey a single exponential distribution; instead, they obey a large number of exponential distributions whose expected values range quasi‐continuously. The corresponding initiation rate thus spans a nearly continuous spectrum. Two mechanisms were speculated to be at work. The ON state is composed of a large number of sub‐states, each with a distinct initiation rate. These sub‐states are defined by specific binding of transcription factors or epigenetic marks and are closely spaced in time, so that they are hard to distinguish or count. Alternatively, in the ON state the promoter switches between a primed state and a mature state that produces a transcript. The mature state depends on the local and time‐varying concentration of Pol II. Such a continuum feature was reported previously (Bothma *et al*., 2014).

The multi‐scale model stresses that transcription is initiated in units of bursts and the bursts are separated with multiple timescales (Tantale *et al*., 2016) (Fig. 2D). Each burst is triggered by a Pol II convoy – a group of Pol IIs (with the number ranging from 1 to 30) successively launches from the promoter within several minutes and then elongates at a rate of ∼4.1 kb/min. With the Mediator at the promoter, the refractory period between two successive bursts obeys an exponential distribution with the expected value of ∼100 s. Such Mediator‐dependent two‐state dynamics have a precondition that the TATA‐box binding protein (TBP, one subunit of TFIID) is at the promoter. Typically, several convoys form and depart in the presence of the TBP. Without the TBP, the promoter enters a non‐permissive period, which does not obey a single exponential distribution; presumably, the non‐permissive periods depend on different extents of promoter methylation or occupancy by histones.

Compared with the simple two‐state model, the ratchet, continuum, and multi‐scale models more accurately describe transcriptional dynamics and provide more insights into transcriptional regulation. However, these models appear to differ substantially from each other. It is unclear to what extent these models are condition specific or reveal the general mechanism of transcription. Interestingly, these models can be unified in an earlier theoretical framework of how the TA operates (Wang *et al*., 2012).

## V. OPERATING PRINCIPLES OF THE EUKARYOTIC TRANSCRIPTION APPARATUS

Transcription is a complex and highly dynamic process (Fuda *et al*., 2009; Vera *et al*., 2016; Cuvier & Fierz, 2017; Teves *et al*., 2018). Thousands of transcription factors have been identified as participating in eukaryotic transcription; the combinatorial interactions among these factors and specific DNA sequences control the activity of Pol II. Yet the kinetics of these interactions are largely beyond technical detection (Gourse & Landick, 2012; Rusk, 2014; Sung *et al*., 2014; Sepúlveda *et al*., 2016). Despite such complexity and difficulty, it has been shown that the eukaryotic TA mostly shares a general architecture (Yudkovsky, Ranish, & Hahn, 2000; Hahn, 2004; Thomas & Chiang, 2006; Kornberg, 2007; Fuda *et al*., 2009). The core promoter of an active gene is often occupied by the scaffold complex (SCF), which is mainly composed of the general transcription factors including TFII‐A, ‐D, ‐E, and ‐H and the Mediator. The transcriptional activator, whose concentration changes with cellular signalling, associates with the enhancer. The enhancer‐bound activator exerts control over transcription initiation *via* the Mediator. Pol II assembles onto the SCF, followed by formation of the transcription preinitiation complex (PIC), then the open complex (OPC), and the elongation complex, which synthesises a transcript (Kornberg, 2005, 2007; Malik & Roeder, 2005).

Based on the general structural organization and conformational changes of the TA, the ensemble and probability theories were exploited to probe how the TA dynamically operates (Wang *et al*., 2012). Theoretical analyses and numerical simulation revealed that, for the TA to orchestrate a reliable response to changing activator concentration, transcripts are essentially generated in units of bursts. It was also proposed that the TA operates as dictated by the following four principles.

**Figure 4:**
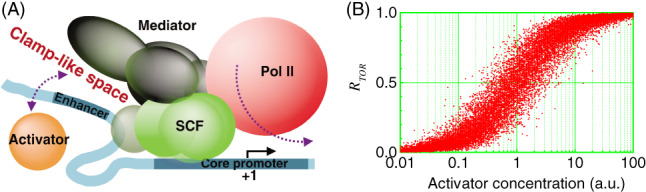
General mechanism of how the transcription apparatus operates in eukaryotes. (A) The Mediator, which is a component of the scaffold complex (SCF), is temporarily tethered in the vicinity of the enhancer, forming a clamp‐like space (C‐space) where activators cycle in and out. With an activator in the C‐space, RNA polymerase II (Pol II) initiates transcription rapidly, leading to a burst of messenger RNAs (mRNAs). (B) With increasing activator concentration, the activators' temporal occupancy rate in the C‐space, *R*_TOR_, rises and saturates in probability. The relationship is a statistical mapping, meaning that slight fluctuations in activator concentration (i.e. extrinsic noise) can be filtered out, thus endowing transcriptional regulation with strong robustness. Shown are simulation results under the condition that activators have cycled for five rounds. Reprinted from Wang *et al*. (2012).

1. Transcriptional activators cyclically bind to and dissociate from the enhancer, with the individual binding time no more than several minutes. Some of those cycling activators act to recruit chromatin modifiers, while some control transcription initiation *via* the Mediator.
2. There temporarily exists a ‘clamp‐like space (C‐space)’ between the enhancer and the Mediator (Fig. 4A), which functions to detect the activator concentration *via* a statistical quantity, *R*_TOR_. The C‐space is relatively stable; it persists for many rounds of activators cycling in and out. *R*_TOR_ is the activators' temporal occupancy rate in the C‐space: *R*_TOR_ = ∑*t*_i_ */T*, where *t*_i_ is the residence time of the *i*‐th (*i =* 1, 2, 3, …, *n*) round of cycling and *T* is the total time corresponding to *n* rounds of cycling. As the activator concentration rises, *n* becomes larger in probability for fixed *T*, and so does *R*_TOR_. In this way, *R*_TOR_ robustly encodes the activator concentration (Fig. 4B). Note that the basic unit of *R*_TOR_ is the individual residence time *t*_i_.
3. The Mediator transfers information from the settled activator to Pol II through allostery. The entry of an activator into the C‐space induces allostery in the Mediator, facilitating Pol II to assemble and initiate transcription. Without the activator, transcription initiation rarely occurs.
4. With an activator in the C‐space, a convoy of Pol IIs is launched, leading to a burst of transcripts. The time interval between two successive initiation events is far shorter than the activator's residence time. Typically, the interval is of seconds. Such intensive initiations together with the Mediator's allostery guarantee that the basic unit of *R*_TOR_ is converted to the number of transcripts.

These principles suggest that transcriptional bursts are separated with multiple timescales and that the initiation rate is continuous during each ON period. The first layer that separates the bursts is controlled by the activator's cycling within the C‐space, manifested as activator–Mediator‐dependent two‐state dynamics. The second layer is determined by the stability of the SCF where the C‐space forms, as marked by the TBP's presence or absence. The third and higher layers depend on the extent to which the promoter is epigenetically modified and recaptured by histones. On the other hand, even with an activator in the C‐space, synthesizing a transcript requires many steps from the SCF, PIC, and OPC, to elongation. These steps do not have identical reaction rates. That is, the intervals between two successive initiation events do not obey an exponential distribution. Given fluctuations in local concentrations of Pol II and general transcription factors such as TFIIF, the initiation rate will range over a wide spectrum. In other words, the multi‐scale and continuum models touch on different aspects of gene transcription, which are shared by various genes to different degrees.

**Figure 5:**
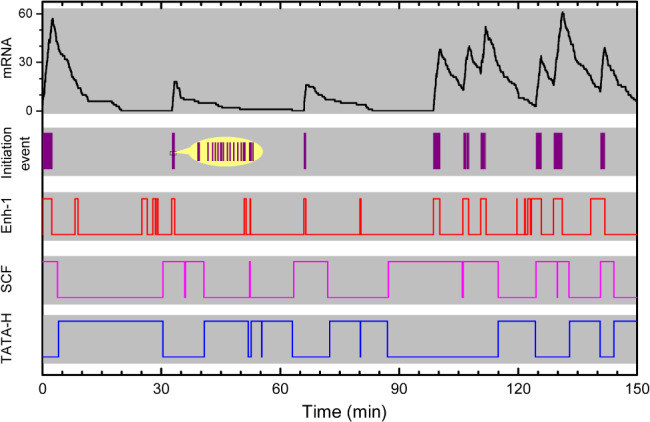
Concurrent multi‐scale and continuum features of transcriptional bursting. Shown are simulation results with the Wang–Liu–Wang (WLW) model. Transcription initiation events are denoted by violet vertical lines, with the inset showing an enlarged view. Enh‐1 denotes whether the enhancer (Enh) is bound by activators, with the upper parts of the line denoting the bound state. SCF and TATA‐H denote whether the TATA‐box in the core promoter region is occupied by the scaffold complex (SCF) and histones (H), respectively. The dynamics of activators cycling in the C‐space, the formation and destruction of the SCF, and occupancy of the core promoter by histones endow the bursts with multiple time scales. The time intervals between two successive initiation events do not obey a single exponential distribution, as shown in the inset.

The above principles are recapitulated by the Wang–Liu–Wang (WLW) model (Fig. 2E), with which simulation results reproduced different profiles of gene expression with the precision reaching the standard deviation of the transcriptional response (Wang *et al*., 2012). In this model, transcription is initiated *via* steps from the SCF, to PIC, to OPC; these steps are tightly controlled by activators *via* the Mediator. The destruction of the SCF may be followed by histone rebinding. Histones can be easily evicted in the presence of enhancer‐bound activators that recruit chromatin remodelling enzymes and modifiers. Notably, numeric simulation with the WLW model reproduces the multi‐scale and continuum features (Fig. 5). Additionally, the WLW model can be expanded to embody the ratchet model if a sufficient number of steps on chromatin modifications are included (Wang *et al*., 2014).

## VI. EFFECTS OF TRANSCRIPTIONAL BURSTING ON EUKARYOTIC CELLULAR SIGNALLING

**Figure 6:**
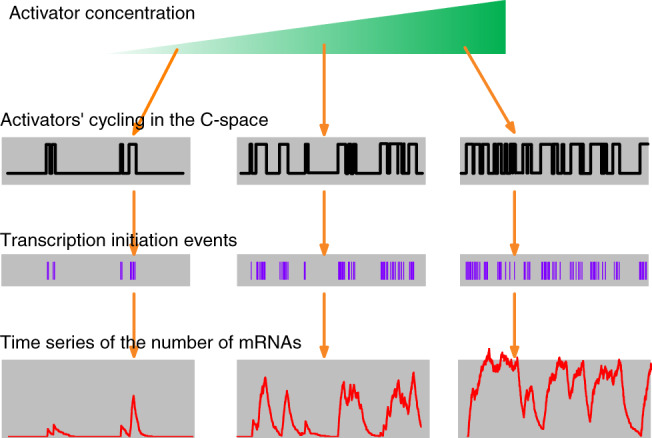
Modulation of transcriptional bursts. Increasing the activator concentration leads to more frequent cycling of activators in the C‐space, with the distribution of residence time unaffected. The residence times shape the occurrence of transcription initiation events, thereby controlling the time series of mRNA number. As a result, the burst frequency, rather than burst size, is subject to modulation by activator concentration. Note that the bursting with high frequency makes it hard to differentiate between two successive bursts.

Given that transcripts are synthesized in episodic bursts, cells must somehow deal with this ‘noisy’ behaviour. This first raises an issue of how the bursting transfers information (Skupsky *et al*., 2010; Dar *et al*., 2012; Molina *et al*., 2013; Corrigan & Chubb, 2014; Senecal *et al*., 2014). As mentioned above, the statistical quantity *R*_TOR_ is exploited by the TA to encode activator concentration and guide transcription initiation (Wang *et al*., 2012); consequently, increasing activator concentration leads to an increase in burst frequency (Fig. 6). That is, the burst frequency represents the strength of regulatory signals. By contrast, burst size is determined by the activators' residence time in the C‐space, irrespective of activator concentration. For a given promoter and constant concentrations of related proteins such as the general transcription factors and Pol II, the burst size obeys a specific distribution. Note that, when detected experimentally, a burst cluster that is composed of two or more closely adjoined bursts could be mistaken as one burst. Because the burst cluster emerges with a larger probability at higher activator concentrations, an inaccurate conclusion might be obtained that the burst size rises with increasing activator concentration (Fig. 6).

Advanced measurements at single‐transcript resolution validated these theoretical predictions. For the *c‐Fos* gene, for example, the burst frequency is modulated by activator concentration, and the burst duration is determined by the duration of activator binding to DNA (Senecal *et al*., 2014). Additionally, the transcription initiation rate during activator–DNA binding will be affected if the strength of the activator's transactivation domain is altered by mutation. The same conclusions were reached for steroid‐receptor‐induced gene transcription (Larson *et al*., 2013). Increasing the activated steroid receptor leads to an increase in burst frequency, whereas the duration and magnitude of individual bursts are not affected. Further analyses on enhancer–promoter communications in living *Drosophila* embryos, mouse, and human cells also confirmed this frequency modulation (Bartman *et al*., 2016; Fukaya *et al*., 2016). Collectively, transcriptional bursting is modulated in a digital manner.

Fluctuations due to bursting can be directly utilized by positive feedback loops in gene regulatory networks to induce the bistability of gene expression and thereby cell differentiation (Kussell & Leibler, 2005; Eldar & Elowitz, 2010; Torres‐Padilla & Chambers, 2014; Buettner *et al*., 2015; Dueck *et al*., 2016). Nevertheless, a second issue arises concerning how the accuracy of cellular signalling is achieved given the fluctuations. Recent studies showed that, after the release of a transcript from DNA, the mature mRNA tends to reside in the nucleus for a long time (Bahar Halpern *et al*., 2015; Battich, Stoeger, & Pelkmans, 2015). mRNAs produced in discontinuous bursts thus accumulate in a pool and are slowly released into the cytoplasm. It was thus suggested that the nuclear retention of mRNAs tends to minimize the burst noise.

## VII. CONCLUSIONS

(1) Genes are transcribed in episodic bursts. This feature is ubiquitous from bacteria to mammalian cells, regardless of whether they are constitutive or inducible genes.

(2) Transcriptional bursts in eukaryotes take place with multiple timescales, and the transcription initiation rate is continuous during each ON period. Such features are consistent with those dictated by four theoretically derived principles that govern how the TA operates dynamically. This in turn suggests that eukaryotic genes likely share similar dynamic principles for TA operation, as they share the same set of general transcription factors.

(3) The ratchet, multi‐scale, and continuum models emphasize different profiles of transcriptional dynamics. These models are applicable to genes of specific categories and can be unified by the WLW model, which depicts the core steps of regulated transcription and can be accommodated to characterize gene specificity. Additionally, conclusions derived from the traditional two‐state model should be treated cautiously.

(4) Transcriptional bursting in eukaryotes allows digital information conversion, by which regulatory signals modulate the burst frequency rather than burst size. For a given TA, the burst size obeys a specific distribution; experimentally, the burst size might be mistaken as subject to modulation since it is hard to differentiate a burst from a burst cluster.

(5) Transcriptional bursting fulfills the accuracy and stochasticity requirements for the transcriptional response. The modulation of burst frequency is an embodiment of the *R*_TOR_ code, which represents an accurate encoding of time‐varying activator concentration; moreover, bursting‐induced fluctuations in mRNA number can be smoothed by nuclear retention of mRNAs. On the other hand, such fluctuations can also be enlarged to achieve sufficient heterogeneity.

(6) Low‐affinity DNA binding sites, unstable protein complexes, and DNA supercoiling can play crucial roles in regulating transcription. Investigating transcriptional dynamics necessitates both live imaging methods with high resolution (Skupsky *et al*., 2010; Suter *et al*., 2011; Evans *et al*., 2012; Friedman, Mumm, & Gelles, 2013; Gebhardt *et al*., 2013; Hocine *et al*., 2013; Kouno *et al*., 2013; Lickwar, Mueller, & Lieb, 2013; Yunger *et al*., 2013; Sidaway‐Lee *et al*., 2014; Annibale & Gratton, 2015; Camunas‐Soler *et al*., 2015; Gocheva *et al*., 2015; Roberts *et al*., 2015; Rybakova *et al*., 2015a; Corrigan *et al*., 2016; Tantale *et al*., 2016) and quantitative computer simulations with appropriate theories and models (Skupsky *et al*., 2010; Suter *et al*., 2011; Wang *et al*., 2012; Maina *et al*., 2014; Choubey, Kondev, & Sanchez, 2015; Stefan *et al*., 2015; Rybakova *et al*., 2015a,*b*; Corrigan *et al*., 2016; Tantale *et al*., 2016). Specifically, integrating diverse sets of data makes it possible to present a coherent dynamic picture of gene transcription in bacteria (Wang *et al*., 2016).

(7) The significance of various bursting patterns from different promoters remains to be addressed. Firstly, unscrambling the bursting patterns paves the way to reveal the dynamics of both the chromatin and the TA. By statistically analysing the Mediator‐ and TBP‐dependent bursting dynamics (such as the burst number, duration, and interval), for example, it can be inferred that the Mediator interacts with the DNA much more transiently than the TBP (Tantale *et al*., 2016; Teves *et al*., 2018). Secondly, it is an open issue why different genes adopt different bursting patterns to realize their functions. In other words, are distinct bursting patterns exploited or accommodated to achieve specific signalling capability?

## VIII. ACKNOWLEDGEMENTS

This work was supported by the Ministry of Science and Technology of the People's Republic of China [2013CB834104], National Natural Science Foundation of China [31361163003, 81421091, and 11175084], and Fundamental Research Funds for the Central Universities [JUSRP11838].
